# Rating the aesthetic results after auricular reconstructive surgery for congenital aural atresia with microtia

**DOI:** 10.1017/S0022215124000914

**Published:** 2024-10

**Authors:** Njima Schläpfer, Livia Papp, Dirk Lehnick, Meike Harder, Daniel Simmen, Thomas Linder

**Affiliations:** 1 Department of Otorhinolaryngology, Head & Neck Surgery, University of Lucerne, Cantonal Hospital of Lucerne, Lucerne, Switzerland; 2 Department of Biostatistics and Methodology, University of Lucerne, Cantonal Hospital of Lucerne, Lucerne, Switzerland; 3 ORL-Zentrum, The Hirslanden Clinic, Zürich, Switzerland

**Keywords:** congenital microtia, ear, esthetics, surgery, plastic, outcome assessment, health care

## Abstract

**Objective:**

This retrospective study aimed to establish a robust rating system for assessing post-operative outcomes in congenital aural atresia patients undergoing auricular reconstruction. The newly introduced EAR scale, a weighted grading system, not only considers anatomical landmarks but also factors such as ear alignment. In addition, the outer-ear cartilage scale and the visual analogue scale (VAS) were introduced. These scales were compared among themselves and against two established scales.

**Methods:**

Nine raters assessed 17 eligible patients who underwent auricular reconstruction between 2001 and 2020.

**Results:**

The study compared inter-rater agreement among scales, with the EAR scale proving the most reliable (Krippendorff's alpha coefficient, *α* = 0.45), outperforming existing measures. The outer-ear cartilage scale and the VAS exhibited lower inter-rater agreement, indicating inferiority in assessing aesthetic outcomes.

**Conclusion:**

The EAR scale emerged as an effective tool for evaluating post-operative outcomes in congenital aural atresia auricular reconstruction.

## Introduction

### Background

Microtia is a congenital hypoplastic malformation of the pinna with a worldwide prevalence of 0.83 to 4.34 per 10,000 births.^1–5^ Males are more than twice as likely to be affected and the condition is usually unilateral (77 per cent to 93 per cent unilateral involvement).^6^ The severity of the malformation may range from slightly smaller subunits of an otherwise completely developed auricle to a completely missing pinna, also called anotia. Microtia is often associated with partial or total atresia of the external auditory canal, as well as malformations of the middle ear. We hereafter refer to the combination of microtia and atresia as congenital aural atresia (CAA).

A widely used classification to describe congenital deformities of the auricle has been published by Weerda and proposes three different grades of dysplasia.^7^ Depending on the severity of dysplasia, congenital ear deformities are challenging regarding both aesthetic and functional reconstructive surgery. The aim of auricular reconstructive surgery of congenital aural atresia is to achieve an aesthetically pleasing ear with restoration of recognisable anatomical landmarks.

At our centre, the reconstruction of congenital aural atresia is a threefold procedure involving the insertion of a rib cartilage framework. It can be combined with functional reconstruction of the acoustic meatus and eardrum in the event of a favourable middle-ear anatomy, or in combination with the implantation of an acoustic implant. In the first session, the auricular rudiments are removed and a rib cartilage framework is placed under the skin. After at least three months, the healed ear framework is lifted off the back of the head and the earlobe is correctly positioned, with a skin graft to cover the exposed wound area behind the reconstructed auricle. Fine-tuning of the reconstructed pinna can be performed subsequently. It involves scar correction, removal of excess skin or modelling of an eventual depression of the auditory canal.

Several reconstructive surgical techniques are available, but few attempts have been made so far to develop an easy-to-use reliable tool to compare post-operative outcomes. Skarzynski *et al*.^8^proposed a weighted 10-point scoring system based on anatomical landmarks, hereafter called the Skarzynski scale. Outcomes are classified into four categories (I = perfect reconstruction, II = complete functional and aesthetic reconstruction, III = satisfactory functional reconstruction, IV = unsatisfactory functional and aesthetic reconstruction).

Another grading system by Sharma *et al*.^9^ uses a weighted 13-point scoring system also based on anatomical landmarks and classification into four categories (poor, average, good, excellent). The helix is the highest weighted anatomical landmark in both scoring systems. While Sharma *et al*.^9^ use anatomical landmarks exclusively, Skarzynski *et al*.^8^ also consider the complete elevation of the helix from the surface of the skin.

Others have used a 12-point scoring system to compare aesthetic outcomes after different types of skin-coverage methods, considering skin colour, thin coverage (convolution), ear size and bilaterally balanced projections.^10^ Constantine *et al*.^11^ compared the reconstruction technique with rib cartilage versus a porous polyethylene implant, using a five-point scale to rate six categories: protrusion, definition, shape, size, location and colour match.

### Objectives

The aim of this study was to develop a new scale for evaluating aesthetic results after auricular reconstructive surgery of congenital aural atresia. Former published grading systems and the analysis of post-operative results after reconstruction surgery at our institution served as a basis for the development of the outer ear and EAR scales. Analysing the appearance of the cartilage framework, we also propose a slightly modified version called the outer-ear cartilage scale. The aim of this scale is to rate the quality of the cartilage framework and to determine putative correlations between the intra-operatively constructed cartilage framework and post-operative outcomes. The overall result is assessed by means of a visual analogue scale (VAS). Neither consideration of the cartilage framework nor the assessment by the VAS have been included in the studies published until now.

## Materials and methods

### Study design

**Figure 1.**
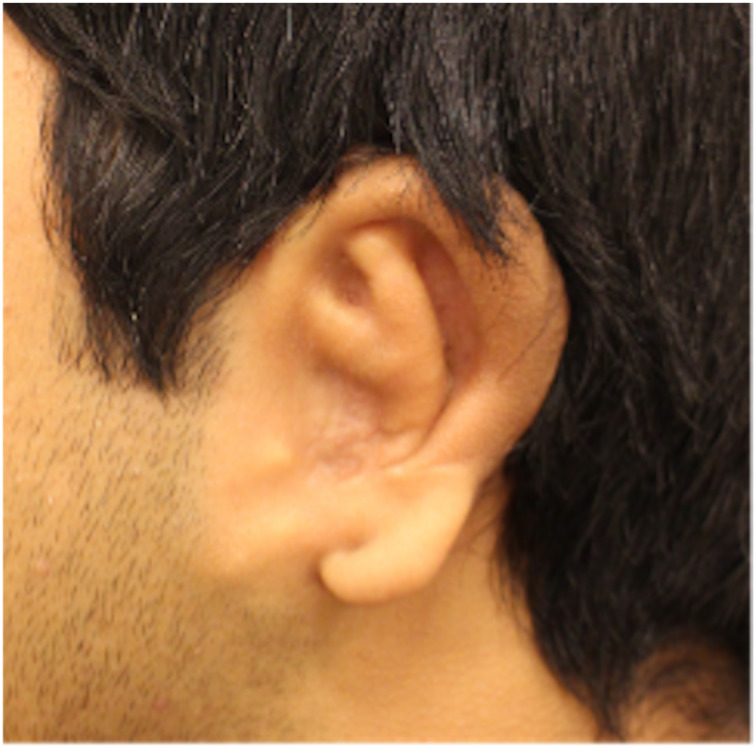
Reconstructed ear.

**Figure 2.**
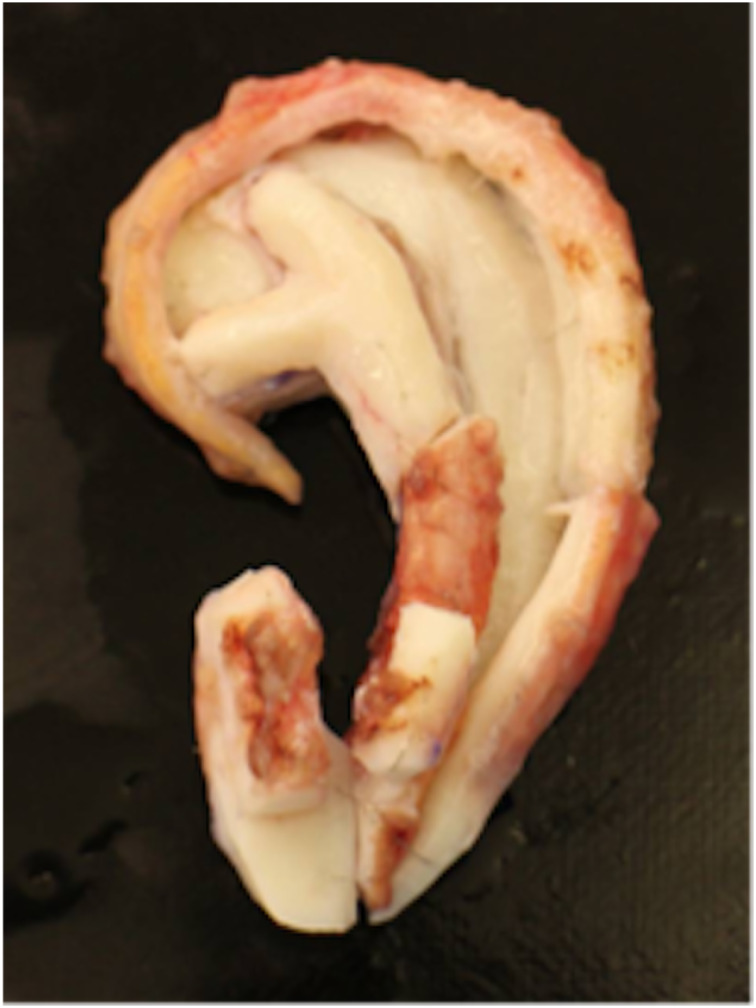
Cartilage framework.

The study was designed as a retrospective data analysis using the EAR scale, the outer-ear cartilage scale and the VAS grade of the aesthetic post-operative outcomes of patients based on photographs of auricular reconstructive surgery of congenital aural atresia and of the cartilage framework (Figures 1 and 2).

### Participants

All consecutive patients with congenital aural atresia treated by auricular reconstructive surgery between January 2001 and December 2020 were assessed for eligibility. Their medical charts and photographs were reviewed. Patients without general consent forms, those who had not undergone all three stages and patients with missing pre- or post-operative photographs were excluded.

**Figure 3.**
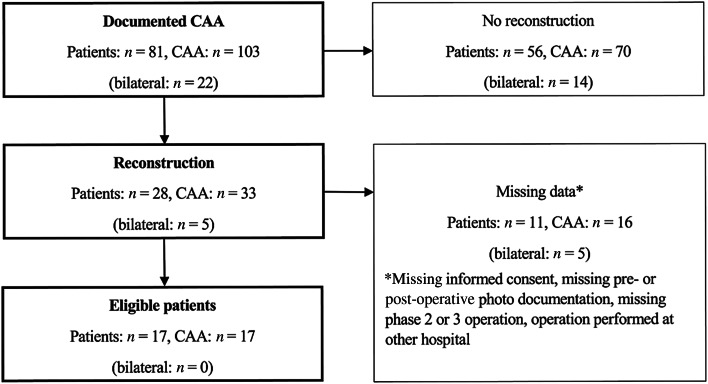
Flow chart of study enrolment: patients with congenital aural atresia treated by auricular reconstruction surgery between 1 January 2001 and 31 December 2020 at the Department of Otorhinolaryngology, Head & Neck Surgeryof the Cantonal Hospital of Lucerne.

Out of the 81 patients identified, 22 presented bilateral congenital aural atresia, resulting in 103 ears with congenital aural atresia. In 70 cases, either no auricular reconstruction had been performed or the patients were too young to undergo surgery. Thirty-three ears were thus treated with auricular reconstructive surgery. Another 16 ear atresia patients could not be evaluated owing to missing or incomplete data. Our final patient population was therefore composed of 17 patients with unilateral CAA (Figure 3). The intra-operative photographs of the cartilage framework were missing for five patients, resulting in 12 patients being assessed by the outer-ear cartilage scale. Informed consent was obtained from all patients included in the study.

### Description of the scales

#### The EAR scale

The EAR scale is a weighted rating scale with a maximum score of 13 points. The helix is weighted with a maximum of four points, the lobulus with a maximum of three points and the anthelix with a maximum of two points. These three anatomical landmarks are thus the most heavily weighted anatomical landmarks. The tragus, cavum conchae and/or external auditory canal, symmetrical ear alignment and symmetrical ear projection (ear–head angle) are weighted with a maximum of one point each (Tables 1 and 2). Depending on the score obtained, a grading system divides the aesthetic result into four categories: a score of 13 points is excellent, 10–12 is good, 5–9 is average and below 5 is poor.

**Table 1.** The EAR scale: weighted grading system for aesthetic outcome after microtia reconstruction

Outcome	Grade (points)
Helix	
– Not present	0
– Present with incomplete rim	3
– Present with complete rim	4
Lobulus	
– Not present	0
– Rudimentary present	1
– Present, but poorly positioned and/or disproportioned	2
– Correctly positioned and well-formed	3
Anthelix	
– Not present	0
– Rudimentary present	1
– Superior and inferior crus visible	2
Tragus	
– Not present	0
– Present	1
Cavum conchae and/or external meatus	
– Not present	0
– Visible/identifiable	1
Symmetrical alignment of ears	
– No/not assessable	0
– Yes	1
Symmetrical auricular projection (ear-to-head angle)	
– No/not assessable	0
– Yes	1
Total (maximum) score	13

**Table 2.** Evaluation of microtia treatment results according to the 13-point and 4-grade EAR scale

Grade	Total score (points)	Outcome
1	13	Perfect reconstruction
2	10–12	Good reconstruction
3	5–9	Average reconstruction
4	0–4	Poor reconstruction

#### The outer-ear cartilage scale

The outer-ear cartilage scale is used to assess the constructed auricular cartilage framework intra-operatively (Tables 3 and 4). It also has a maximum score of 13 points. Like the EAR scale, the helix is weighted with a maximum of four points, the lobulus with a maximum of three points and the anthelix with a maximum of two points. The tragus and cavum conchae are weighted with a maximum of one point. Unlike the EAR scale, the overall impression is also analysed and scored with a maximum of two points. Ear alignment and ear projection can obviously not be rated when only rating the cartilage framework. A score of 13 points is excellent, 10–12 points is good, 5–9 points is average and 4 or below is poor.

**Table 3.** Outer-ear cartilage scale: weighted grading system for cartilage framework in microtia reconstruction

Outcome	Grade (points)
Helix	
– Not present	0
– Present with incomplete rim	3
– Present with complete rim	4
Lobulus	
– Not present	0
– Rudimentary present	1
– Partially formed, clearly distinguishable	2
– Fully formed	3
Anthelix	
– Not present	0
– Rudimentary present	1
– Superior and inferior crus present	2
Tragus	
– Not present	0
– Present	1
Cavum conchae	
– Not present	0
– Well distinguishable	1
Total (maximum) score	11

**Table 4.** Evaluation of cartilage framework according to the 22-point and 4-grade outer-ear cartilage scale

Grade	Total score (points)	Outcome
1	20–22	Perfect reconstruction
2	13–19	Good reconstruction
3	5–12	Average reconstruction
4	0–5	Poor reconstruction

#### The visual analogue scale

**Figure 4.**
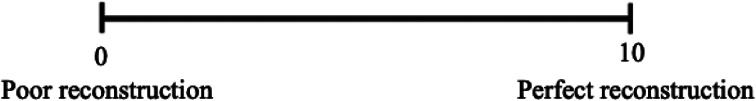
Visual analogue scale.

In the VAS, the overall impression of the auricle is evaluated on a scale from 0 (indicating poor reconstruction) to 10.0 (indicating perfect reconstruction) (Figure 4). The scale is displayed on a 10-cm measuring line, where each centimetre represents 1 rating point. The overall impression is indicated by a mark along the line. The rating is read off to one decimal. A score of 9.0–10.0 means excellent reconstruction, 6.0–8.9 is good, 3.0–5.9 is average and 0–2.9 is poor.

### Measurement

The EAR scale, the outer-ear cartilage scale, the VAS and the Skarzynski and Sharma scales^8,9^ were applied to the set of intra- and post-operative photographs. Nine physicians of different educational status in our ENT department conducted the ratings independently: three experienced ENT surgeons who operate on congenital aural atresia, four experienced ENT surgeons not involved in reconstructive surgery, one ENT resident and one medical student. The sequence of post- and intra-operative photographs was presented randomly to each rater.

### Statistical methods

All statistical evaluations were completed using Stata (version 17.0, StataCorp, College Station, TX, USA). Categorical variables were summarised by absolute and relative frequencies. Quantitative variables were analysed using descriptive statistics. To assess the consistency of agreement between scales, intraclass correlations and corresponding 95 per cent confidence intervals (CIs) were calculated, based on a mixed-effects model with a fixed effect for scale and random effects for rater and patient. The analysis of the inter-rater agreement was performed using Krippendorff's alpha coefficient (*α*) and its 95 per cent CI.

This article is written in accordance with the Strengthening the Reporting of Observational Studies in Epidemiology guidelines.^12^ The authors assert that all procedures contributing to this work comply with the ethical standards of the Swiss ethics committee (Project ID 2021-01815) and with the Helsinki Declaration of 1975, as revised in 2008.

## Results and analysis

### Baseline patient characteristics and demographics

**Figure 5.**
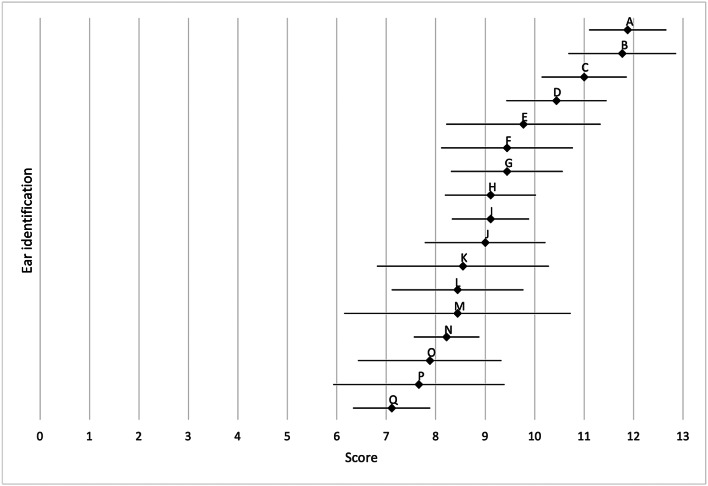
EAR scale: mean (♦) and standard deviation (—) of post-operative outcomes (*n* = 17). Each letter stands for one patient.

**Figure 6.**
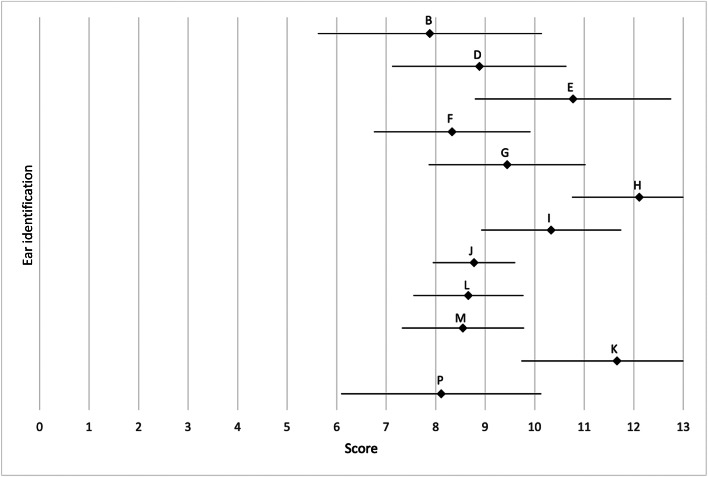
OEC scale: mean (♦) and standard deviation (—) of the intra-operative cartilage framework (*n* = 12). Each letter stands for one patient.

Thirteen out of 17 patients were male (76 per cent) and 4 were female (24 per cent). The median age at first surgery was 13 years (minimum, 9 years; maximum, 23 years). Ten patients underwent surgery on the left ear (59 per cent) and 7 on the right ear (41 per cent). Additional canaloplasty with meatoplasty was performed in 13 cases (76 per cent). Figures 5 and 6 show the median and standard deviations of the rated post-operative outcome score of each patient for the EAR scale (*n* = 17) and the outer-ear cartilage (OEC) scale (*n* = 12).

### Inter-rater reliability

The inter-rater agreement was highest with Krippendorff's *α* of 0.45 (95 per cent CI 0.23–0.67) for the EAR scale. The Skarzynski scale showed a coefficient of 0.42 (95 per cent CI 0.22–0.62). The coefficient for the Sharma scale was only slightly lower at 0.40 (95 per cent CI 0.26–0.55) (Table 5).

**Table 5.** Interrater agreement

Interrater agreement	Krippendorff's alpha coefficient, *α* (95% CI)
EAR scale	0.45 (0.23–0.67)
OEC scale	0.34 (0.15–0.53)
Visual analogue scale	0.38 (0.24–0.53)
Sharma scale	0.40 (0.26–0.55)
Skarzynski scale	0.42 (0.22–0.62)

CI = confidence interval

### Correlations

The highest intraclass correlation was obtained with an intraclass correlation value of 0.70 (95 per cent CI 0.60–0.77) when comparing the rating scales of Sharma and Skarzynski. The EAR scale was correlated with the Skarzynski scale with an intraclass correlation value of 0.65 (95 per cent CI 0.54–0.73) and with the Sharma scale with an intraclass correlation value of 0.57 (95 per cent CI 0.45–0.66). The correlation between the EAR scale and the VAS was practically equivalent, with an intraclass correlation value of 0.57 (95 per cent CI 0.45–0.67). The Sharma scale and the VAS were correlated with an intraclass correlation value of 0.55 (95 per cent CI 0.43–0.65), and the intraclass correlation value for the correlation between the Skarzynski scale and the VAS was 0.49 (95 per cent CI 0.36–0.60). The OEC scale for the cartilage framework was poorly correlated with all other scales, with an intraclass correlation value ranging from 0.05 to 0.15 (Table 6).

**Table 6.** Intraclass correlation with 95% confidence interval

Intraclass correlation consistency of agreement	EAR scale	OEC scale	VAS	Sharma scale	Skarzinsky scale
EAR scale	1	0.10 (−0.09–0.29)	0.57 (0.45–0.67)	0.57 (0.45–0.66)	0.65 (0.54–0.73)
OEC scale		1	0.15 (−0.04–0.33)	0.22 (0.04–0.39)	0.05 (−0.14–0.23)
VAS			1	0.55 (0.43–0.65)	0.49 (0.36–0.60)
Sharma scale				1	0.70 (0.60–0.77)
Skarzynski scale					1

VAS = visual analogue scale

We summarised the correlation of all scales evaluating the post-operative photographs (the EAR scale, the VAS, the Sharma scale and the Skarzynski scale, while excluding the OEC scale) in an overall agreement. The intraclass correlation value for these four scales was 0.59 (95 per cent CI 0.51–0.66). When excluding the VAS, the intraclass correlation value increased to 0.63 (95 per cent CI 0.55–0.71).

## Discussion

Cosmetic and functional reconstruction of congenital aural atresia remains challenging. A thorough analysis of the final outcome, a retrospective review of each surgical step and a score result should help the surgical team to improve their learning curve. Close collaboration between the otologist and the ENT plastic surgeon is essential to achieve the best possible outcome for the patient. Our EAR scale provides a simple, quick way of assessing post-operative results. Existing rating scales mainly rely on the presence or absence of anatomical landmarks of the ear to assess the final result. The EAR scale not only takes the presence or absence of anatomical landmarks into account, but also scores them from an aesthetic point of view. It also evaluates the alignment and projection of the ears, which are important factors in the assessment of the aesthetic result that were not included in formerly published grading systems.

- Cosmetic and functional reconstruction of congenital aural atresia poses surgical challenges, with limited tools comparing post-operative outcomes
- The EAR scale, the OEC scale and a visual analogue scale were used to assess aesthetic outcomes
- The EAR scale not only considers anatomical landmarks, but also evaluates ear alignment and projection, aspects overlooked in previous grading systems
- Factors other than the cartilage framework by itself lead to differences in final outcomes
- The EAR scale proved effective in evaluating post-operative outcomes in congenital aural atresia auricular reconstruction

Statistical interpretation was limited owing to the small number of only 12 patients. In summary, all three post-operative assessment scales (the EAR scale, the Skarzynski scale and the Sharma scale) correlate well with each other, measuring approximately the same features. The strongest correlation was found between the Skarzynski and Sharma scales, as both rate solely the anatomical landmarks without considering the alignment or projection of the ears, unlike the EAR scale.

The inter-rater reliability was measured to determine the level of consensus of various physicians. The inter-rater agreement was highest in the EAR scale compared with the other grading systems (Krippendorff's *α* of 0.45, moderate correlation). The Skarzynski scale showed an equal inter-rater agreement with Krippendorff's *α* of 0.42.

Unexpectedly, the OEC scale correlated poorly with the EAR scale, the Sharma scale and the Skarzynski scale. Other factors, such as wound healing, skin texture, circumscribed post-operative infections or foreign body reactions to the suture material, also have an influence on the post-operative outcome, therefore the aesthetics of the cartilage framework alone do not allow a firm conclusion to be drawn about the post-operative aesthetic outcome. The variety of malformations and therefore the limited standardised reconstruction technique of the cartilage framework and soft tissue may also explain the lack of correlation.

The VAS did not correlate either with the EAR scale, the OEC scale, the Sharma scale and the Skarzynski scales. It does not seem to be a suitable tool for assessing an aesthetic outcome, given the subjectivity associated with using a visual analogue scale.

Our pilot study involved various ENT physicians. We did not take into account the patients’ own opinions or those of lay persons. However, the results should please the patients and their families, an issue that has not yet been addressed.

## Conclusion

This study has shown that the EAR scale is an easily applicable tool for rating post-operative aesthetic outcomes after congenital aural atresia reconstructive surgery because it assesses more aesthetically relevant aspects than formerly published rating systems. Further research should seek to validate the scale by applying it in a larger patient and rater population. Furthermore, it would be interesting to compare the reconstructed ear with the healthy ear in unilateral cases. It would also be interesting to compare the correlation between physician-rated outcome and patient-rated outcome. Additionally. the EAR scale can also be used to evaluate results after artificial framework implantations, which have not been implemented at our centre yet.
